# Bacterial Targets of Antibiotics in Methicillin-Resistant *Staphylococcus aureus*

**DOI:** 10.3390/antibiotics10040398

**Published:** 2021-04-07

**Authors:** Harshad Lade, Jae-Seok Kim

**Affiliations:** Department of Laboratory Medicine, Kangdong Sacred Heart Hospital, Hallym University College of Medicine, Seoul 05355, Korea; harshadlade@gmail.com

**Keywords:** methicillin-resistant *Staphylococcus aureus* (MRSA), antibiotic targets, cell wall, peptidoglycan synthesis, protein synthesis, teichoic acid, lipid II

## Abstract

Methicillin-resistant *Staphylococcus aureus* (MRSA) is one of the most prevalent bacterial pathogens and continues to be a leading cause of morbidity and mortality worldwide. MRSA is a commensal bacterium in humans and is transmitted in both community and healthcare settings. Successful treatment remains a challenge, and a search for new targets of antibiotics is required to ensure that MRSA infections can be effectively treated in the future. Most antibiotics in clinical use selectively target one or more biochemical processes essential for *S. aureus* viability, e.g., cell wall synthesis, protein synthesis (translation), DNA replication, RNA synthesis (transcription), or metabolic processes, such as folic acid synthesis. In this review, we briefly describe the mechanism of action of antibiotics from different classes and discuss insights into the well-established primary targets in *S. aureus*. Further, several components of bacterial cellular processes, such as teichoic acid, aminoacyl-tRNA synthetases, the lipid II cycle, auxiliary factors of β-lactam resistance, two-component systems, and the accessory gene regulator quorum sensing system, are discussed as promising targets for novel antibiotics. A greater molecular understanding of the bacterial targets of antibiotics has the potential to reveal novel therapeutic strategies or identify agents against antibiotic-resistant pathogens.

## 1. Introduction

Antibiotics remain the primary agents in the treatment of bacterial infections, and their use has reduced patient mortality and increased life expectancy. However, continuous evolution and dissemination of antibiotic-resistant pathogens pose a serious threat to the effective treatment of bacterial infections [1]. To maximize the efficacy of antibiotics currently in clinical use and to develop new antibiotics with novel mechanisms of action, it is essential to identify potential bacterial targets. Examination of bacterial target diversity in the context of antibiotics currently in clinical use has revealed that not all essential biochemical processes represent antibiotic targets in bacteria. Fortunately, most antibiotics selectively target and inhibit one or more bacterial biochemical processes, e.g., cell wall (peptidoglycan) synthesis [2,3], protein synthesis (translation) [4,5,6], DNA replication [7,8], RNA synthesis (transcription) [9,10], and folic acid biosynthesis [11,12], while few antibiotics act by interfering with ion channels and bacteriolysis [13].

The Gram-positive bacterial pathogen *Staphylococcus aureus* is the cause of serious infections, particularly since the emergence of antibiotic-resistant methicillin-resistant *S. aureus* (MRSA). MRSA is a notorious pathogen that causes hospital-acquired infections worldwide and is associated with high morbidity and mortality [14]. Although most MRSA isolates are susceptible to standard-of-care and last-line drug vancomycin, and to the newly introduced antibiotics daptomycin, linezolid, tedizolid, ceftaroline, and quinopristin/dalfopristin, there is a risk of the emergence of strains resistant to these antibiotics. It is therefore important to identify novel agents that inhibit well-established targets and/or essential cellular processes of MRSA, as inhibitors of these targets have the potential to be developed into new classes of antibiotics.

Herein, we discuss the known antibiotic targets in *S. aureus* and summarize the molecular mechanisms that are predicted to dictate bacterial death. Furthermore, the antibiotic classes currently in clinical use that target one or more bacterial biochemical processes are described. 

## 2. Antimicrobial Resistance in *S. aureus*

*S. aureus* is a commensal bacterium in humans and is transmitted in both community and healthcare settings [15]. It is the leading cause of invasive or complicated infections, including bacteremia, pneumonia, endocarditis, skin and soft tissue infections, osteoarticular infections, and osteomyelitis [16,17]. *S. aureus* becomes life-threatening when it evades the host immune system, crosses the epithelial barrier, and gains access to deeper tissues, such as blood, heart valves, the gastrointestinal tract, dermis, or bones [18,19]. People are at higher risk of *S. aureus* infections when they are admitted to hospitals, have surgery or are fitted with implantable medical devices, or when they come into contact with *S. aureus*-infected patients [14]. The data from Emerging Infections Program (EIP) MRSA population surveillance (2005–2016) and Premier and Cerner Electronic Health Record databases (2012–2017) describing the trends in incidence of hospital-onset and community-onset MRSA and MSSA bloodstream infections showed that *S. aureus* bloodstream infections account for significant morbidity and mortality in the United States. In 2017, an estimated 119,247 *S. aureus* bloodstream infection with 19,832 associated deaths occurred in the United States [20]. Further, the data from bacterial culturing and drug susceptibilities collected in Japan Nosocomial Infection Surveillance (JANIS) showed that an estimated 17,157 *S. aureus* bloodstream infection-associated deaths out of the whole population (126.8 million) occurred in Japan in 2017 [21]. Among them, cases attributed to MRSA accounted for 4224 (24.6%). The problem is exacerbated by the emergence and rapid spread of MRSA, which is resistant to almost all known β-lactam antibiotics [22]. It has been reported that MRSA strains are associated with an increase in mortality of more than 60% when compared with methicillin-susceptible *S*. *aureus* (MSSA) [23]. Antibiotics in clinical use are becoming increasingly ineffective against MRSA as antimicrobial resistance spreads worldwide [24]. 

**Figure 1:**
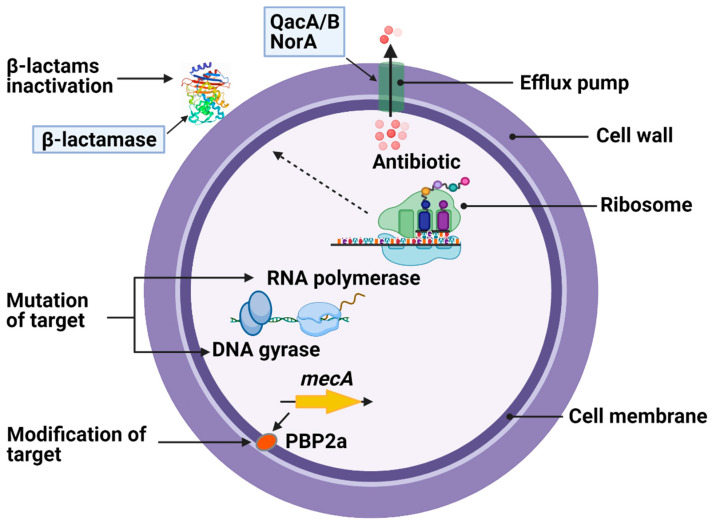
Molecular mechanisms of antibiotic resistance in *Staphylococcus aureus*. Antimicrobial resistance occurs due to the mutation or modification of antibiotic targets, inactivation of β-lactam antibiotics by β-lactamase, a reduction in membrane permeability, or increased activity of efflux pumps.

The selective pressure exerted by exposure to various antibiotics has led to the emergence of multidrug-resistant MRSA strains. The detailed mechanism of this antimicrobial resistance has appeared in recent reviews and is briefly illustrated in Figure 1 [25,26]. MRSA can either develop through the acquisition of determinants by horizontal gene transfer of mobile genetic elements (MGEs) [27]; from transposons and the staphylococcal cassette chromosome element (SCC*mec*) [28]; from chromosomal mutations that alter drug binding sites on molecular targets; or by increasing expression of endogenous efflux pumps [29]. The QacA/B, NorA, and Smr multidrug efflux proteins are present on the *S. aureus* cell membrane [29,30]. MRSA harboring *qacA* and *qacB* genes has been found to be associated with increased resistance to non-β-lactam antibiotics and chlorhexidine tolerance [31].

Resistance genes for various antibiotics have been identified in both MSSA and MRSA, such as penicillin (*blaZ*), erythromycin (*ermC* and *ermA*), clindamycin (mainly *ermC* encoded inducible resistance), tetracyclines (*tetK* and *tetL*), and trimethoprim (*dfrA* and *dfrK*) [32,33]. MRSA has acquired resistance to all β-lactam antibiotics through two distinct mechanisms: (i) the production of penicillin-binding protein 2a (PBP2a), a transpeptidase that is highly resistant to inhibition by β-lactam antibiotics and can maintain peptidoglycan crosslinking, thus allowing the strain to survive in the presence β-lactam [34,35,36]; and (ii) the production of β-lactamases, which hydrolyze the amide bond of the four-membered β-lactam ring, rendering the antibiotic inactive before it gets to the PBP target [37,38].

PBPs are membrane-anchored enzymes responsible for the final step of bacterial cell wall synthesis and are primary targets for β-lactam antibiotics [39]. Altered PBPs are the major cause of β-lactam antibiotic resistance, especially among Gram-positive cocci. MRSA resistance to β-lactam antibiotics is mediated by PBP2a [34,40]. Furthermore, the aux (auxiliary factors) and fem (factors essential for methicillin resistance) contribute to PBP2a-mediated β-lactam resistance [41,42]. In MRSA, PBP2a (PBP2’) is encoded by the *mecA* gene located on the mobile genetic element SCC*mec*, along with its regulators *mecR1* and *mecI* [43,44]. The majority of healthcare-associated (HA)-MRSA strains are linked to SCC*mec* types II or III, whereas community-associated (CA)-MRSA to SCC*mec* types IV [45,46]. Recently, the *mec*C encoded homolog of PBP2a (designated PBP2c by European Committee on Antimicrobial Susceptibility Testing; EUCAST) from the *S. aureus* strain LGA251 has been described, which differs in β-lactam binding characteristics [47]. PBP2a is also observed in other Gram-positive bacteria, but the DNA sequences and mechanisms by which they confer resistance to β-lactam antibiotics are different [48,49,50]. For instance, PBP2a in the β-lactam-resistant Gram-positive bacterial pathogen *Streptococcus pneumoniae* is bifunctional, polymerizing glycan chains (glycosyltransferase activity) and crosslinking them with peptide bonds (transpeptidase activity) [50,51]. However, PBP2b and PBP2x of *St. pneumoniae* are the primary resistance determinants for different classes of β-lactam antibiotics [51].

Although PBP2a is the major determinant of β-lactam resistance in MRSA, β-lactamase production is another most common resistance mechanism to β-lactam antibiotics [37,38]. The acquisition of *bla*Z gene in *S. aureus* encodes β-lactamases that are able to inactivate β-lactam antibiotics (penicillin and cefazolin) and render them from reaching their target proteins [52]. Staphylococcal β-lactamase enzyme is plasmid-mediated and can be non-inducible or inducible with antibiotics [53]. In Gram-negative bacteria, β-lactamases are the most important resistance determinant for β-lactam antibiotics, with multiple enzymes residing on MGEs in different bacterial pathogens, including *Escherichia coli* and *Pseudomonas aeruginosa* [54,55]. The role of β-lactamases in resistance to β-lactam antibiotics among Gram-negative bacteria has been described extensively elsewhere [54,56,57,58].

Beyond resistance to β-lactams, MRSA is often resistant to other classes of antibiotics currently in clinical use. The majority of HA-MRSA are resistant to non-β-lactams, such as aminoglycosides, lincosamides, fluoroquinolones, and macrolides [59], but resistance to standard-of-care drug vancomycin and linezolid is uncommon [60]. The emergence of vancomycin-resistant *S. aureus* (VRSA) is the most worrying *S. aureus* genetic adaptation due to the reliance on this antibiotic in the treatment of MRSA infections [15]. VRSA has been shown to emerge through plasmid transfer of the *vanA* operon from vancomycin-resistant *Enterococcus faecalis* [61,62]. Although *vanA* is known as a major determinant of VRSA, the further understanding of other resistance mechanisms could identify novel targets in MRSA. For instance, *S. aureus* exhibits vancomycin resistance due to mutations that alter genes encoding core components of the cell membrane (*mprF*), cell wall synthesis and autolysis (*yycH*), and teichoic acid synthesis (*dltA*) [63]. *mprF, yycH*, and *dltA* mutations additionally confer cross-resistance of VRSA to daptomycin [64,65,66]. *yycH* is involved in the *WalKR* cell wall regulatory operon controlling cell wall synthesis and autolysis [67]; thus, mutation of *yycH* results in reduced WalRK activation, impaired cell wall turnover, and, ultimately, reduced vancomycin efficacy. The *mprF* mutation causes alterations in membrane surface charge, leading to resistance to the positively charged antibiotic daptomycin [68]. Mutation in the putative synthase domain of *mprF*, along with enhanced *mprF* expression, elevated biosynthesis of lysylphosphotidylglycerol, increased positive membrane surface charge, and reduced daptomycin surface binding, are all factors contributing to *S. aureus* resistance to daptomycin [69].

## 3. Treatment of MRSA Infections

β-lactams remain an important class of antibiotics for the treatment of *S. aureus* infections, but almost all β-lactams are ineffective against common MRSA, in particular those implicated in skin and soft tissue infections (SSTIs) [70]. Antibiotic selection depends on bacterial susceptibility, patient characteristics, and infection site. MRSA responds to certain antibiotics of each antibiotic class, but MRSA prevalence in hospitals, antibiotic resistance, and disease burden mean it is often necessary to use last-line or new antibiotics to treat persistent infections. The standard-of-care drug vancomycin remains the preferred antibiotic for the treatment of serious MRSA infections, but its effectiveness is restricted by persistent or recurrent bacteremia, nephrotoxicity, and the emergence of non-susceptible strains [24]. Alternative antibiotics, such as linezolid and daptomycin, are comparable to vancomycin in effectiveness [71,72,73,74]. Currently, vancomycin, teicoplanin, arbekacin, and linezolid are used as therapeutic agents for the treatment of MRSA infections [75]. Furthermore, daptomycin and tedizolid were launched in 2011 and 2018, respectively, for the treatment of MRSA infections in Japan [75]. A new cephalosporin antibiotic, ceftaroline fosamil, has been approved in Europe [76] and the USA [77] for the treatment of complicated SSTIs and community-acquired (CA) pneumonia. 

To combat the emergence of further antibiotic-resistant strains, it is necessary to understand the cellular processes of *S. aureus* that are not targeted by current antibiotics in clinical use. Current antibiotic development pipelines mainly focus on the combination of known antibiotics as a strategy to limit the development of antibiotic resistance [78]. As of March 2021, among 43 antibiotics currently in global clinical development, 18 had the potential to treat *S. aureus* infections (Table 1) (The Pew Charitable Trusts; URL: https://www.pewtrusts.org/en/research-and-analysis/data-visualizations/2014/antibiotics-currently-in-clinical-development, accessed on 12 March 2021). Most of these antibiotics have broad-spectrum activity against various bacterial pathogens and would potentially address many, but not all, resistant bacteria. However, some of these antibiotics may fail to get approval, and resistance will eventually develop to any approved antibiotics.

Another strategy suggested for the treatment of MRSA infections is the targeting of so-called virulence factors rather than targeting bacterial growth or viability [79]. The use of antivirulence compounds may not exert the selective pressure on bacteria that leads to the emergence of antimicrobial resistance [80]. Furthermore, phage therapy, the use of bacteriophage viruses that kill specific bacteria through lytic activity, is being considered as there is little or no human toxicity from such viruses, and there is a highly diverse selection of natural phages available, suggesting that complete resistance would be difficult to evolve [81,82]. For instance, the bacteriophage lysin PlySs2 is undergoing Phase 3 clinical trials as an addition to standard-of-care antibiotics for the treatment of patients with *S. aureus* bacteremia, including endocarditis (https://clinicaltrials.gov/ct2/show/NCT04160468, accessed on 12 March 2021). PlySs2, originally derived from a *Streptococcus suis* phage, has an *N*-terminal cysteine-histidine-dependent amidohydrolases/peptidases domain and a C-terminal SH3b cell wall-binding domain [83]. The precise enzymatic activity of PlySs2 has not been defined, but preliminary studies suggest that it acts as a d-Ala-Gly endopeptidase to cleave stem peptide cross-bridges [84]. Furthermore, phage therapy requires comprehensive knowledge of the genetic factors that influence host range.

## 4. Antibiotic Targets in *S. aureus*

Antibiotics are used to eradicate the infecting bacterial pathogen from its host in the shortest possible treatment period. The targets for antibiotics are absent in eukaryotic cells, including those of humans, which means that antibiotics are specific for bacteria and do not damage human cells [85]. Most antibiotics in clinical use target essential bacterial processes or metabolic pathways, leading to the cessation of growth or cell lysis. Furthermore, antibiotics can disrupt cell wall synthesis, cell membrane permeability, or inhibit essential enzymes or apparatuses involved in the synthesis of DNA, RNA, or protein. Antibiotics are most effective against actively growing bacterial cells and ineffective in eradicating persistent infections (slow-growing or dormant bacteria). Different classes of antibiotics exist with distinct bacterial targets or modes of action, such as β-lactams, cephalosporins, glycopeptides, lipopeptides, aminoglycosides, oxazolidinones, macrolides, tetracyclines, rifamycin, fluoroquinolones, sulfonamides, and sulfamethoxazole-trimethoprim. Antibiotics of different classes, their primary target in *S. aureus*, and their mechanisms of action are shown in Table 2.

**Figure 2:**
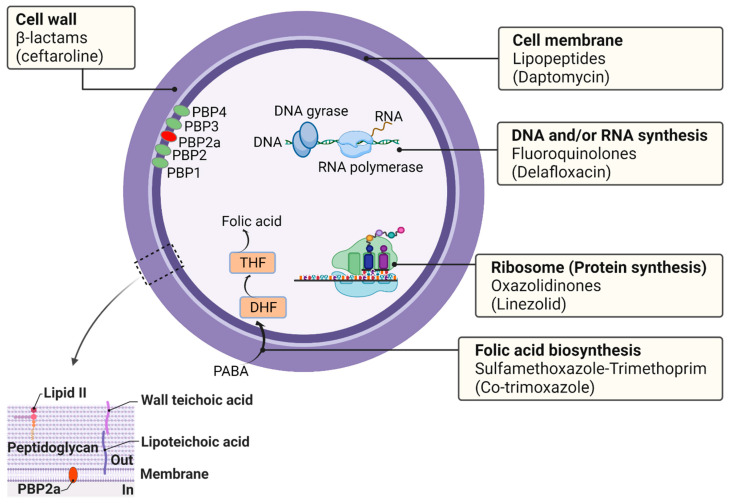
The primary targets of antibiotics in *S. aureus*. Antibiotics can inhibit the growth of bacteria by targeting their cell wall, cell membrane, DNA replication, RNA synthesis, or ribosomes (protein synthesis), leading to bacterial death. Antibiotics can also inhibit folic acid biosynthesis through a pathway involving para-amino-benzoic acid (PABA), tetrahydrofolate (THF), and dihydrofolate (DHF) as precursors of folic acid.

In principle, there are five primary targets for antibiotics in *S. aureus*: (i) the cell wall, (ii) the cell membrane, (iii) DNA and/or RNA synthesis, (iv) ribosomes (protein synthesis), and (v) folic acid biosynthesis (folate metabolism) (Figure 2). 

### 4.1. Cell Wall

The bacterial cell wall is a highly complex multicomponent structure and has been validated as a potential target for antibiotics [114]. The cell wall maintains the shape, size, and integrity of bacteria by regulating the pressure potential (Ψ_P_) and mechanochemical properties of cells [115,116]. It also provides mechanical strength in counteracting intracellular osmotic pressure [117,118]. A core component of the bacteria cell wall is peptidoglycan, a macromolecule composed of glycan strands connected (crosslinked) through flexible species-specific peptide bridges, making a mesh-like structure that surrounds the cell [119,120,121]. The glycan strands are composed of alternating amino sugars *N*-acetylglucosamine (GlcNAc) and *N*-acetylmuramic acid (MurNAc) connected through β-(1,4)-glycosidic bonds [122]. Each MurNAc residue is connected to a short amino acid chain of L-alanine, D-glutamine, L-lysine, and D-alanine with pentaglycine cross-bridges in Gram-positive bacteria (e.g., *S. aureus*) or L-alanine, D-glutamic acid, *meso*-diaminopimelic acid, and D-alanine in Gram-negative bacteria (e.g., *E. coli*). Gram-positive bacterial peptidoglycan matrix is generally thick (30–100 nm) and contains many layers protecting a single cytoplasmic membrane, whereas Gram-negative bacteria have a thinner peptidoglycan matrix and a periplasmic space [123]. Peptidoglycan is a unique component of the bacterial cell and is absent in eukaryotes; thus, it is a safe target for antibiotics.

Staphylococcal peptidoglycan is characterized by pentaglycine cross-bridges that are crosslinked between adjacent wall peptides by PBPs to provide mechanical strength and flexibility during all stages of bacterial growth [121,124,125,126]. The inhibition of peptidoglycan biosynthesis or the disruption of peptidoglycan integrity results in the cessation of cell growth, suggesting that peptidoglycan assembly is a potential target for antibiotics [127].

PBPs are required for cell wall biosynthesis and maintenance and thus are a key target of the β-lactam antibiotics. PBPs are peptidase enzymes located in the cell membrane that catalyze the crosslinking of adjacent stem peptides to synthesize the peptidoglycan backbone [128]. *S. aureus* has four native PBPs: PBP1, PBP2, PBP3, and PBP4. MRSA contains a fifth PBP, namely PBP2a, a transpeptidase encoded by the *mecA* gene [129]. β-lactam antibiotics act on the enzyme PBPs by binding to the D-Ala-D-Ala dipeptide of the peptidoglycan. High-level resistance to β-lactam antibiotics in MRSA is due to the production of PBP2a, an enzyme that provides transpeptidase activity to allow cell wall biosynthesis in the presence of β-lactams.

**Figure 3:**
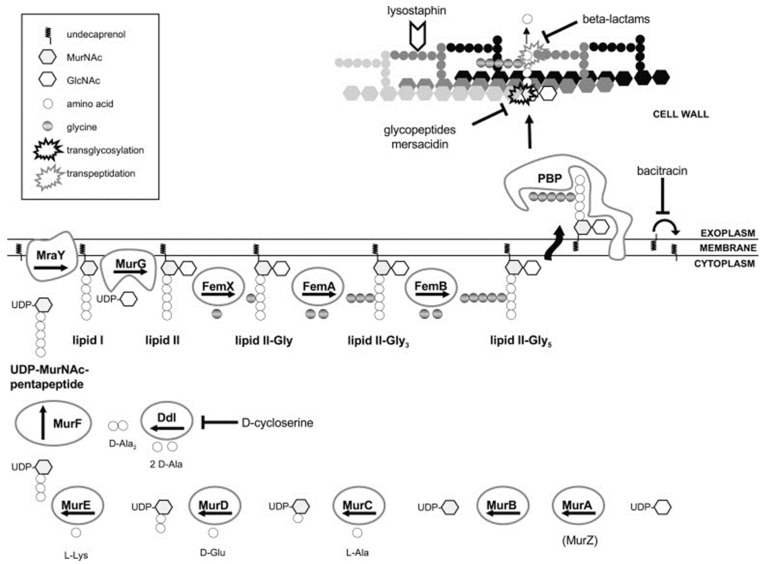
Schematic representation of the peptidoglycan synthesis and targets of cell wall active antibiotics in *S. aureus*. The inhibition of enzymatic activity is indicated by blocked arrows. UDP: uridine-diphosphate, MurNAc: *N*-acetylmuramic acid, GlcNAc: *N*-acetylglucosamine. Adapted from reference [132] with the publisher’s permission.

β-lactams and glycopeptide antibiotics inhibit bacterial cell wall synthesis by blocking or disrupting peptidoglycan biosynthesis (Figure 3) [130]. The new β-lactam class cephalosporin antibiotics have been designed to target PBP2a of MRSA. For instance, ceftaroline, a fifth-generation cephalosporin, binds to PBP2a transpeptidase with high affinity, inhibiting bacterial cell wall synthesis [87,88]. Ceftaroline binds to an allosteric site in PBP2a of *S. aureus*, leading to increased sensitization to the antibiotic [131]. The glycopeptide class antibiotic vancomycin binds to the D-Ala-D-Ala termini moieties of the peptidoglycan precursor lipid II, preventing the incorporation of *N*-acetylmuramic acid (NAM)/*N*-acetylglucosamine (NAG) peptide subunits into growing peptidoglycan chains and consequent transpeptidation catalyzed by PBP2 and PBP2a [91,92], causing an alteration in cell membrane integrity, permeabilization, and bacterial death [3].

The bacterial cell wall serves as a primer for its own biosynthesis. Thus, several antibiotics targeting different stages of cell wall biosynthesis have been reported. The lipopeptide class antibiotic daptomycin-Ca^2+^ complex has recently been shown to target cell wall biosynthesis in *S. aureus* by forming a tripartite complex with lipid II and phosphatidylglycerol, killing the cell [93]. *S. aureus* cell wall contains substantial amounts of teichoic acid, which plays a crucial role in cell division and growth [133] and is, thus, considered a promising target for antibiotics. 

### 4.2. Cell Membrane

The bacterial cell membrane is an essential component of the cell, serving as a selective barrier for the permeation of molecules and ions from the extracellular environment (cellular homeostasis), and is involved in metabolic energy transduction [123]. Differences between prokaryotic and eukaryotic membrane architecture and lipid composition allow the targeting of bacterial membranes by antibiotics [134]. The bacterial cell membrane is characterized by negatively charged phospholipids, such as phosphatidylglycerol, lipoteichoic acids, and lipopolysaccharides. Bacterial cytoplasmic membranes comprise different ionic molecules, such as proteins, glycoproteins, lipoproteins, phospholipids, lipopolysaccharides, peptidoglycan, and teichoic acids, involved in electrostatic interaction with host cell surfaces [135]. The presence of membrane potential and ionic molecules makes the cell membrane one of the primary targets for antibiotics against bacteria. Importantly, cell membranes are an attractive target in non-growing bacterial cells (persisters) [136]. 

Many classes of peptide antibiotics, such as glycopeptides (e.g., dalbavancin), lipopeptides (e.g., daptomycin), and cyclopeptides (e.g., lysobactin and pristinamycin), with targets located in the bacterial cytoplasmic membrane, have been approved for the treatment of bacterial infections [137,138]. Cell membrane-active antibiotics are typically toxic to mammals due to low selectivity, but the success of the lipopeptide class antibiotic daptomycin has triggered renewed interest in membrane-active antimicrobials [139]. Daptomycin showed bactericidal activity against non-growing bacterial cells with rapid killing kinetics [139]. Many antimicrobial peptides with a rigid binding site can bind to lipid components of the bacterial cell membrane without toxicity to host cells [140]. For example, daptomycin possesses high selectivity for the *S. aureus* cell membrane. Daptomycin complexes with Ca^2+^ to form oligomers [94] that insert daptomycin into the cytoplasmic membrane upon binding to phatidylglycerol head groups [95,96], causing membrane depolarization, permeabilization, ion leakage (notably K^+^), and rapid cell death [97,98].

### 4.3. DNA and/or RNA Synthesis

Bacterial DNA topoisomerase II (DNA gyrase), which introduces negative supercoiling into chromosomal DNA, and DNA topoisomerase IV, which promotes chromosome decatenation after replication, are attractive targets for antibiotics due to sequence homology in their corresponding subunits and substantial overlap in their 3-dimensional protein structures. Both enzymes comprise two subunits arranged as a heterotetramer, and targeting these enzymes inhibits the control of supercoiling within the cell, resulting in impaired DNA replication [7,141]. Several antibiotics in clinical use target both enzymes, including the fluoroquinolone class antibiotic delafloxacin. Fluoroquinolones are the most successful synthetic antimicrobials that target nucleic acid biosynthesis in bacteria, preferentially targeting topoisomerase IV in Gram-positive bacteria and DNA gyrase in Gram-negative bacteria [8].

Rifampicin is a broad-spectrum antibiotic that specifically targets prokaryotic RNA polymerases. It acts on bacterial DNA-dependent RNA polymerase, leading to suppression of RNA synthesis and bacterial cell death [9,10]. Rifampicin binds in a pocket of the RNA polymerase β subunit within the DNA/RNA channel, preventing transcription by blocking elongation of the 5′ end of the RNA transcript, leading to the inhibition of protein synthesis [10]. 

### 4.4. Ribosomes (Protein Synthesis)

Bacterial ribosomes are one of the main targets for antibiotics [142,143]. Ribosomes are the site of protein biosynthesis in all living cells and are central to protein synthesis, converting mRNA into corresponding polypeptide chains [144,145]. The bacterial ribosome is a 70S ribonucleoprotein complex composed of a large 50S and a small 30S subunit [144,145,146]. The 50S ribosomal subunit includes 23S and 5S rRNAs, which bind aa-tRNA, catalyze peptidyl transfer, and regulate the elongation process. The 30S subunit includes 16S rRNA, which binds mRNA and initiates protein synthesis. Protein synthesis can be divided into four main steps: initiation, elongation, termination, and recycling. The first two steps are targets of many antibiotics [147,148]. Initiation involves the formation of a 70S ribosome from 50S and 30S subunits around the mRNA and the initiator tRNA, typically fMet-tRNA. This process is controlled by three prokaryotic initiation factors: IF1, IF2, and IF3. During elongation, amino acids are added to the growing peptide chain in a stepwise manner, which is believed to be the heart of protein synthesis.

Ribosome-targeting antibiotics bind to rRNA sequences without direct protein involvement [149,150,151]. These ribosomal antibiotics interfere with protein biosynthesis by different mechanisms: the inhibition of peptide bond formation, the inhibition of protein exit from the 50S subunit tunnel, the inhibition of tRNA binding, and the inhibition of subunit association, disassociation, and turnover [150,151]. Additionally, the biogenesis of ribosome subunits has been suggested as a potential target for antibiotic inhibition [152,153]. It is reported that many clinically used antibiotics specifically target bacterial ribosomes [151], targeting different stages of bacterial protein synthesis based on their binding site in the ribosome or by binding to other protein factors associated with protein biosynthesis. 

Aminoglycosides are a class of natural and semisynthetic antibiotics that exert broad-spectrum bactericidal activity by interacting with the ribosome and inhibiting protein synthesis [154]. The aminoglycosides alter protein synthesis by stimulating miscoding errors and by binding to bacterial ribosomes, inhibiting their translocation on mRNA [154,155]. They are used synergistically in combination with either glycopeptides or β-lactam to treat complicated *S. aureus* infections [156]. Arbekacin, an aminoglycoside antibiotic, is approved for the treatment of pneumonia and sepsis caused by MRSA [157,158]. Macrolide class antibiotics, including azithromycin, have a specific binding site in domain V of the 23S rRNA. Azithromycin interacts with the 23S rRNA, inhibiting translation by blocking aminoacyl-tRNA, peptidyl-tRNA, or the peptide exit tunnel in a nascent peptide sequence-specific fashion [6]. Oxazolidinones (e.g., linezolid and tedizolid) are the new class of synthetic antibiotics that inhibit protein synthesis by binding to the ribosome. Linezolid interferes with the initiation of the ternary complex formation between tRNA^fMet^, mRNA, and the 70S ribosome, thereby halting translation [4].

### 4.5. Folic Acid Biosynthesis (Folate Metabolism)

**Figure 4:**
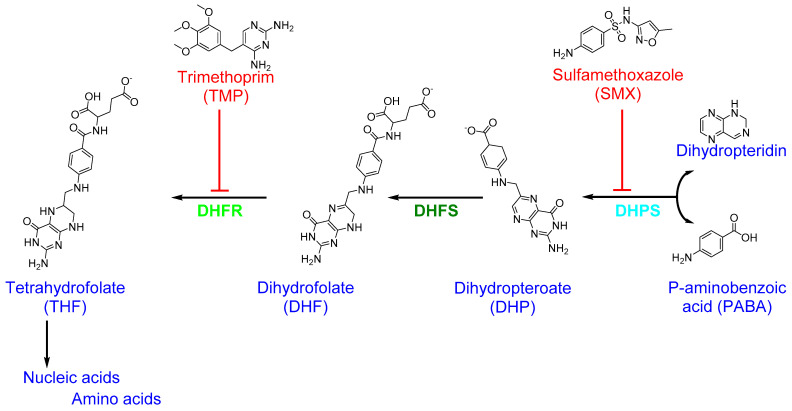
Folic acid biosynthesis pathway in bacteria. The key enzymes involved in folic acid metabolism are labeled in green, dark green, and turquoise while antibiotics that inhibit them are shown in red.

The folic acid (folate) pathway is a potential target for antibiotics since folic acid is essential for cell division as it is required for the biosynthesis of nucleic acid [159]. The catalytic activity and structure of all enzymes in the folic acid pathway, and the differences between bacteria and human cells, are well characterized [108,160]. Based on the metabolic differences in folic acid biosynthesis between bacteria and humans, several drugs that target this pathway have been validated for clinical use [159,161]. The key enzymes involved in bacterial folic acid biosynthesis and the antibiotics that inhibit them are shown in Figure 4.

Dihydrofolate reductase (DHFR), an enzyme that catalyzes nicotinamide adenine dinucleotide phosphate (NADPH)-dependent reduction in dihydrofolate (DHF) to tetrahydrofolate (THF) in bacteria and eukaryotes cells, is a common antibiotic target in folic acid biosynthesis [162]. DHFR is a crucial enzyme for folic acid biosynthesis in bacteria and is required for the continued synthesis of nucleic acid [11]. Trimethoprim (TMP; 2,4-diamino-5-(3′,4′,5′-trimethoxybenzyl)pyrimidine), a diaminopyrimidines class synthetic drug used for the treatment of UTIs, inhibits bacterial DHFR and the conversion of DHF to THF [11,112]. TMP is specific to *S. aureus* DHFR (SaDHFR) than to human DHFR, which leads to preferential inhibition of bacterial folic acid synthesis [163]. However, *S. aureus* has developed resistance to TMP through the acquisition of plasmid-derived DHFR or a Phe98Tyr point mutation in chromosomal SaDHFR [163]. Moreover, a transposon-encoded *dfrA* gene, which encodes a TMP-resistant type S1 DHFR, has been shown to mediate TMP resistance [164,165]. Indeed, *dfrA* (also known as *dfrS1*), *dfrD*, and *dfrG* genes have all been detected in livestock-associated (LA)-MRSA, whereas Tn*4003*-associated *dfrS1* is widespread in *S. aureus*, and *dfrG* is observed frequently in MRSA [166,167].

A combination of two antibiotics, sulfamethoxazole (SMX; 3-(p-aminophenyl sulfonamido)-5-methylisoxazole) and TMP, known as co-trimoxazole, has been used to treat bacterial urinary tract infections (UTIs) and uncomplicated SSTIs caused by MRSA [168,169,170]. While TMP itself is bactericidal, its combination with SMX is synergistically effective against MRSA [169]. The two agents, SMX and TMP, act as antimetabolites by inhibiting the enzymes involved in the bacterial synthesis of folic acid. SMX is a sulfanilamide drug structurally analogous to para-aminobenzoic acid (PABA), a substrate critical for the biosynthesis of folic acid [171]. SMX binds to bacterial dihydropteroate synthase (DHPS), which catalyzes the conversion of PABA to dihydropteroate (DHP) in the process of THF formation [168]. DHPS inhibition leads to defective thymidine biosynthesis and, consequently, slows or blocks folic acid biosynthesis [108]. In general, the SMX–TMP combination works to block two consecutive steps in nucleic acid and protein biosynthesis that are essential for bacterial growth and division [113]. The widespread use of SMX–TMP to treat staphylococcal infections in children has resulted in the development of antimicrobial resistance in both MSSA and MRSA [172,173,174].

## 5. Other Promising Targets

Although drug discovery research is still targeting the pathways described above using additional structural data on antibiotic target proteins to aid the design of novel antimicrobial agents, for example, components of other fundamental cellular processes have also been suggested as targets for novel antibiotics in the treatment of *S. aureus* infections, such as the teichoic acid, aminoacyl-tRNA synthetases, the lipid II cycle, auxiliary factors of β-lactam resistance, two-component systems, and the accessory gene regulator quorum-sensing system. 

### 5.1. Teichoic Acid Synthesis

Teichoic acid (TA) glycopolymers are a major component of the cell walls of Gram-positive bacteria, including staphylococci, and represent up to 60% of cell wall dry weight [175]. TAs comprise polyribitol-phosphate polymers crosslinked to MurNAc residues of peptidoglycan, covered with D-alanine and GlcNAc residues [176]. There are two types of TAs: lipoteichoic acid (LTA) and wall teichoic acid (WTA). TAs are either anchored in the plasma membrane as LTA or covalently linked to peptidoglycan as WTA. In *S. aureus*, the LTA backbone of 1,3-linked glycerol phosphate repeat units is bound to gentiobiosyl-diacylglycerol (Glc2-DAG), while WTAs comprise 1,5-linked ribitol-phosphate subunits anchored to peptidoglycan via a disaccharide linkage unit.

The LTA in *S. aureus* is a poly-glycerophosphate polymer attached to the outer surface of the cell membrane [123]. LTA has several roles in cell envelope physiology, including regulation of cell autolysis, coordination of cell division, and adaptation to environmental growth conditions [177]. LTA is further modified with substituents, including glycosyl groups and D-alanine, to change cellular function. The biosynthetic pathways for LTA and WTA have been well defined in *S. aureus* [177,178]. For instance, D-alanylation is the most well studied pathway by which DltA loads the carrier protein DltC with D-alanine [179,180]. It is shown that the addition of D-alanine ester by dltABCD operon regulates cell autolysis during cell wall remodeling [181], PBP4 localization [182], and confers resistance to antimicrobial peptides [180,183] and antibiotic daptomycin [184].

**Figure 5:**
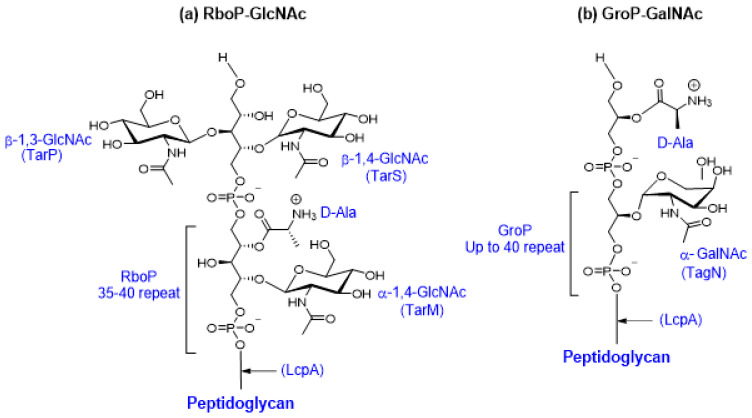
Structure of *S. aureus* wall teichoic acids (WTAs). (**a**) The ribitol-phosphate (RboP) WTA in the majority *S. aureus* lineages comprises a polymerized RboP backbone modified with α-1,4-GlcNAc, β-1,4-GlcNAc, β-1,3-GlcNAc, and D-alanine residues. (**b**) The glycerol-phosphate (GroP) WTA of the phylogenetically isolated *S. aureus* ST395 lineage comprises a polymerized GroP backbone, modified with α-linked GalNAc by the glycosyltransferase TagN.

WTAs are linear structures composed of ribitol-phosphate or glycerol-phosphate subunits polymerized by phosphodiester bonds [185]. In *S. aureus*, WTAs are major components of the cell wall, where they contribute substantially to the integrity of the cell envelope [185]. The major clonal lineages of human pathogenic *S. aureus* produce cell wall-anchored poly-ribitol-phosphate (RboP) WTA surface polymers with up to 35-40 repeating units substituted with GlcNAc or D-alanine [186] (Figure 5a). The RboP WTA is synthesized in the cytoplasm; modified by glycosyltransferases TarM, TarS, and/or TarP intracellularly; exported; and attached to the peptidoglycan layer. The phylogenetically distinct *S. aureus* ST395 lineage, associated with livestock animal MRSA, produces a unique poly-glycerol-phosphate (GroP) WTA modified with α-linked *N*-acetylgalactosamine (GalNAc) by the glycosyltransferase TagN (Figure 5b) [187,188]. WTAs regulate cell division, mediate host colonization, protect the cell from osmotic stress, and mask susceptible peptidoglycan bonds [178,189]. Importantly, modifications in the WTA provide *S. aureus* resistance to cationic antimicrobial peptides (AMPs) by the shifting membrane charge and the electrostatic repulsion of cationic peptides [190,191].

Both LTA and WTA in *S. aureus* have been suggested as potential targets for therapeutic and preventative strategies, such as phage therapies, antibody therapies, and anti-*S. aureus* vaccines, since humans have no homologous structures and TAs play a crucial role in bacterial survival, resistance to AMPs and antibiotics, host defenses, colonization to the surface, cell division and growth, cell shape maintenance, and regulation of ion homeostasis [175,177,178,192]. Furthermore, TAs in *S. aureus* are key targets of the host immune system due to their exposure and high abundance on the bacterial surface [185]. The presence of either LTAs or WTAs on the cell surface is necessary for the initiation of cell division in *S. aureus* [193]. Tunicamycin selectively inhibits TarO, the first enzyme in the WTA biosynthetic pathway, and sensitizes MRSA to β-lactam antibiotics, even if the PBP2a transpeptidase is expressed [194]. This sensitization was attributed to defects in the synthesis of peptidoglycan (a stress-bearing cell wall polymer of a disaccharide of NAM and NAG) due to possible localized alterations of either PBP2 or PBP2a. The inhibition of WTA synthesis blocks the cooperative action of PBPs that underlie β-lactam resistance in MRSA [195]. Although WTAs are not requisite for survival *in vitro,* inhibiting the biosynthesis pathway at a late stage is bactericidal. Targocil inhibits a late step in the WTA pathway by targeting TarG, a transmembrane component of the ABC transporter (TarGH) that exports WTA to the cell surface [196]. However, modifications in both LTA and WTA have been shown to affect their functions, resulting in changes in resistance to antibiotics and cationic AMPs [181,185]. For instance, *S. aureus* with no D-alanine ester in either TA has been shown to exhibit three-fold-increased sensitivity to vancomycin and is more susceptible to the staphylolytic enzyme lysostaphin due to reduced autolytic activity [181]. Indeed, inhibition of both LTA and WTA is lethal in *S. aureus* [197,198].

### 5.2. Aminoacyl-tRNA Synthetases

The aminoacyl-tRNA synthetases are essential and universal ‘housekeeping’ enzymes in protein biosynthesis that catalyze the esterification of amino acids to their cognate tRNA molecules in a highly specific two-step reaction [199,200,201]. Aminoacyl-tRNA synthetases recognize amino acids, ATP, and cognate tRNA, and facilitate high-fidelity protein synthesis [202]. Inhibition of any one of these stages leads to the accumulation of uncharged tRNA, which attaches to ribosomes, resulting in an interruption in the elongation of the polypeptide chain and inhibition of protein synthesis [203]. Aminoacyl-tRNA synthetases specific for each of the 20 amino acids have been reported [204,205].

As an essential component of protein translation in bacteria, aminoacyl-tRNA synthetase enzymes represent a potential target for antibiotics to treat bacterial infections. For instance, phenylalanine tRNA synthetase (PheRS) is a unique aminoacyl-tRNA synthetase enzyme in *S. aureus* essential for protein synthesis [206]. *S. aureus* PheRS is composed of two alpha subunits (PheS) and two larger beta subunits (PheT). Mupirocin (pseudomonic acid A), an antibiotic produced by *Pseudomonas fluorescens*, targets a specific type of aminoacyl-tRNA synthetase enzyme (aaRS), leading to the inhibition of protein synthesis and MRSA growth [207,208]. Since PheRS plays a crucial role in *S. aureus* protein synthesis, there is great potential for the development of novel drugs targeting this enzyme to treat MRSA infections.

### 5.3. Lipid II Cycle

Lipid II is a membrane-anchored cell wall precursor of peptidoglycan that is essential for cell wall biosynthesis in bacteria and is, thus, a target of a variety of different classes of antibiotics [209]. The lipid II cycle is a core pathway in cell wall biosynthesis in Gram-positive bacteria and is involved in shuttling peptidoglycan subunits across the cytoplasmic membrane [210]. MraY and MurG bind UDP-MurNAc-pentapeptide and UDP-GlcNAc to the membrane-associated carrier undecaprenyl phosphate (UP) to produce lipid I and lipid II intermediates, respectively [211]. These lipid II cycle reactions represent the rate-limiting step in cell wall biosynthesis, and different antibiotics act by inhibiting its progress. The inhibition of the lipid II cycle in bacteria is achieved by either targeting the activity of enzymes involved, e.g., PBPs (inhibited by β-lactams), MurG (inhibited by ramoplanin), MraY (inhibited by tunicamycin), or by direct sequestration of the intermediate substrates of the lipid II cycle, e.g., UPP (sequestered by bacitracin), UP (sequestered by friulimicin), or lipid II (sequestered by vancomycin and nisin) [209,210].

The lipid II cycle is a potential target for antibiotics in Gram-positive bacteria, including *S. aureus*. Generally, antibiotics targeting lipid II cause sequestration of the precursors from PBPs and the subsequent inhibition of transglycosylation and transpeptidation. A variety of different classes of antibiotics, including the clinically relevant glycopeptide vancomycin, target the lipid II cycle. Vancomycin forms hydrogen bonds with the D-Ala-D-Ala termini moieties of lipid II, leading to conformational alterations that prevent the incorporation of NAM/NAG peptide subunits into the growing peptidoglycan chain and consequent transpeptidation [91,92]. This alters bacterial cell membrane integrity, membrane permeability, and ultimately leads to bacterial death [3].

### 5.4. Auxiliary Factors in β-Lactam Resistance

A major determinant of β-lactam antibiotics resistance in MRSA is the decreased affinity of β-lactams for PBP2a [34,36], an enzyme that catalyzes DD-transpeptidation of peptidoglycan. However, the full expression of PBP2a requires auxiliary factors, which assist PBP2a in conferring β-lactam resistance. Thus, inhibition of these auxiliary factors can increase the susceptibility of MRSA to β-lactam antibiotics [212].

Several auxiliary genes, identified as a prerequisite for PBP2a-mediated β-lactam resistance by transposon screens, have been shown to be involved in peptidoglycan precursor synthesis or cell wall turnover [41,213,214]. Furthermore, many auxiliary factors are involved in cell wall biosynthesis and regulation, such as PBP4, which synthesizes peptidoglycan [215]; Fem peptidyltransferases, which add a penta-glycine stem to lipid II [216]; GlcNAc-1-P-transferase TarO, which initiates the biogenesis of WTA [194]; WTA GlcNAc glycosyltransferases TarS and TarP [217]; the polymerizing division protein FtsZ [218]; and the two-component system VraSR [219]. Recently, auxiliary factor A (auxA, SAUSA300_0980) and B (auxB, SAUSA300_1003) have been shown to be central for LTA stability, and inhibitors of these factors can resensitize MRSA to β-lactam antibiotics [212]. Auxiliary factor PrsA plays a role in posttranscriptional maturation of PBP2a, and deletion of PrsA affects oxacillin resistance in different SCC*mec* strains with decreased membrane-associated PBP2a, without affecting *mecA* mRNA levels [213].

### 5.5. Two-Component Systems

Bacterial signal transduction mechanisms known as two-component systems (TCSs) are the primary pathways by which bacteria adapt to changes in the external environment. A prototypical bacterial TCS consists of a transmembrane sensor histidine kinase and a cognate response regulator [220]. The sensor kinase plays a key role in extracellular stimuli recognition and intracellular autokinase activity modulation, leading to the transfer of its phosphoryl group to the cognate response regulator, which modulates downstream gene expression [221]. The core genome of *S. aureus* harbors 16 TCS, while a subtype of MRSA strains contains an additional TCS, the SCC*mec* element (https://www.ncbi.nlm.nih.gov/Complete_Genomes/SignalCensus.html, accessed on 12 March 2021), which is responsible for methicillin resistance [222]. The roles of TCSs in *S. aureus* cellular processes and pathogenesis have been described previously [223]. Briefly, *S. aureus* TCSs have been shown to be involved in antibiotic resistance and the response to cell wall damage (VraRS, GraXRS, and BraSR) [224,225,226,227]; virulence, including biofilm formation (AgrCA and SaeRS) [223,228,229,230]; cell wall synthesis, autolysis, and cell death (WalRK, ArlSR, and LytSR) [231,232,233]; bacterial respiration, nitrate metabolism, and fermentation (SrrBA, NreCAB, and AirRS) [234,235,236]; and nutrient sensing and metabolism (HssRS, KdpDE, and PhoRP) [237,238,239,240]. Importantly, SaeRS, which is composed of the sensor kinase SaeS and response regulator SaeR, controls the production of more than 20 *S. aureus* virulence factors. AgrCA and SaeRS are primarily involved in *S. aureus* biofilm formation through phenol-soluble modulin (PSM) production and the suppression of extracellular protease synthesis, respectively [241]. GraXRS determines *S. aureus* resistance to host innate immune barriers [242]. WalRK is essential for *S. aureus* growth and regulates *lytM*, *atl*, and *ssaA*, while resistance to the last-line antibiotic vancomycin is conferred by VraRS (the vancomycin resistance regulator/sensor) by altering cell wall metabolism [63,224,243]. Many VraRS-regulated genes, such as *pbp2*, *sgtB*, *murZ*, *fmtA*, and *tcaA*/*tcaB*, are associated with peptidoglycan synthesis [244,245].

**Figure 6:**
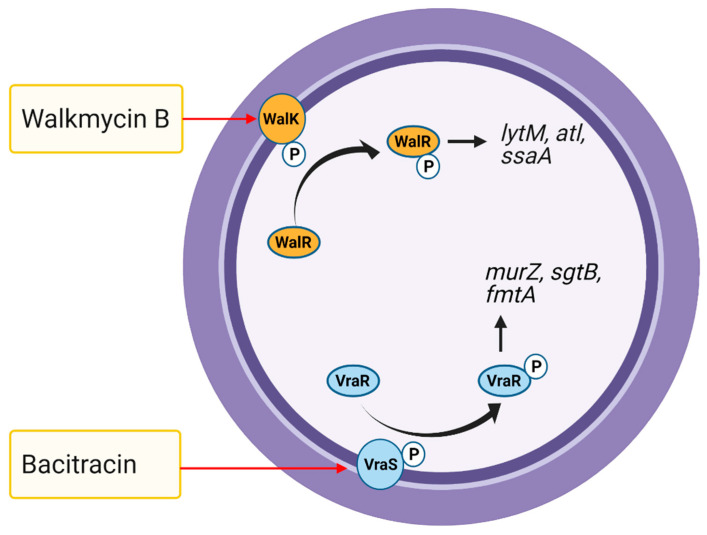
The WalRK and VraRS two-component signal transduction systems (TCSs)-mediated *S. aureus* response to antimicrobial agents. The effect of walkmycin B (WalK autophosphorylation inhibition) and bacitracin (affect VraRS by uncoupling energy required for ATP synthesis) on *S. aureus* is shown.

TCSs are promising targets for novel antibiotics as the genes encoding both the histidine kinase and response regulator are only present in bacterial genomes and not mammals [246]. The essential bacterial growth regulator WalRK and vancomycin resistance regulator/sensor VraRS have been considered attractive drug targets against *S. aureus,* including VRSA [243]. Antibiotics generally target bacterial proteins that perform essential functions, whereas TCS-specific drugs target upstream regulatory functions rather than downstream activities. For instance, the drugs target VraRS function by uncoupling the energy required for ATP synthesis [247]. Thus, drugs that act on TCSs essential for bacterial growth and antibiotic resistance may be promising antibiotics against MRSA [248,249]. For example, walkmycin B (di-anthracenone) specifically targets WalK, a histidine kinase essential for *S. aureus* growth, by inhibiting WalK autophosphorylation (Figure 6) [248]. In response to bacitracin, histidine kinase VraS activates and phosphorylates its response regulator VraR, thereby causes changes in the expression of cell wall synthesis genes (Figure 6) [250]. Norlichexanthone reduces CA-MRSA strain USA300 toxicity to human neutrophils and biofilm formation by directly interfering with AgrA binding to its DNA target and by repressing genes regulated by SaeRS [241].

### 5.6. *The Accessory Gene Regulator* Quorum Sensing System

The accessory gene regulator (*agr*) quorum sensing system is a well-studied major virulence regulatory element in *S. aureus*, which constitutes an extended TCS with sensor histidine kinase AgrC and the DNA-binding response regulator AgrA [251,252,253]. The *agr* system in *S. aureus* regulates the production of pore-forming toxins (PFTs), PSMs, biofilm formation, and heterogeneous resistance [254]. These virulence factors contribute to disease pathogenesis, tissue injury, and failure of treatment, making the *S. aureus agr* system a primary target for the development of antivirulence therapies [79]. Previous drug development programs have provided a wide range of small-molecule ligands for quorum sensing-mediated receptor histidine kinases (RHKs), including AgrC in staphylococci [252,255]. Targeting *agr* quorum sensing does not inhibit growth *in vitro* but mitigates virulence factor production and function, which enhances infection and provokes host damage. Additionally, targeting quorum sensing virulence factors has been shown to exert less selective pressure, which is essential for the development of resistance, than conventional antibiotics [256,257,258]. Given its broad impact on virulence factor production in *S. aureus*, the *agr* quorum sensing system has potential as a target for antivirulence therapies [259,260].

The *agr* system consists of two divergent transcriptional units, termed RNAII and RNAIII, driven by promoters P2 and P3, respectively [261]. The RNAII transcript contains a set of four genes, *agrB*, *agrD*, *agrC*, and *agrA* (*agrBDCA*), which are required for successful quorum sensing [261]. *agrB* encodes a membrane protein associated with translocation and modification of AgrD; *agrD* encodes an auto-inducing peptide (AIP) precursor octapeptide; *agrC* encodes a membrane receptor protein of the AIP signal, and *agrA* encodes the AgrA response regulator that activates transcription. The *agr* system requires secretion and sensing of AgrD as an AIP, which activates the receptor histidine kinase AgrC upon reaching threshold extracellular concentrations [262]. Activated AgrC phosphorylates the response regulator AgrA, which in turn activates transcription at both the P2 and P3 promoters of the *agr* operon, resulting in the expression of RNAIII [228]. This increases the production of various cytolytic virulence factors, including PFTs and PSMs.

**Figure 7:**
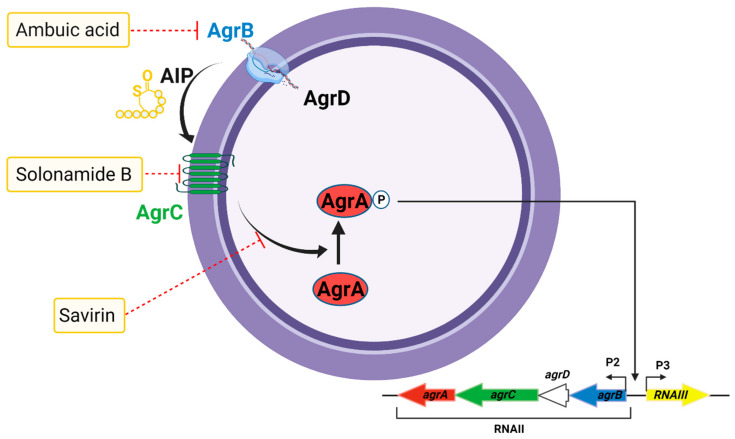
Accessory gene regulator (agr) quorum sensing system in *S. aureus* as a target for the inhibition of virulence factor production. The agr system regulates the production of pore-forming toxins (PFTs) and phenol-soluble modulins (PSMs), which are responsible for disease pathogenesis and tissue injury. Several agents can target the *agr* system to inhibit virulence factor production, including ambuic acid (inhibition of auto-inducing peptide (AIP) production), solonamide B (inhibition of AIP activation of AgrC), and savirin (inhibition of AgrA downstream of AIP sensing).

Although quorum sensing inhibitors lack the characteristics of classical antibiotics, targeting the *agr* quorum sensing system can suppress any step in AIP production, sensing, and subsequent transcriptional activation of the P2 and P3 promoters, as well as the PSM promoter [259,263]. Infection models have revealed that disruption of the timing of *agr* activation leads to reduced infection [264]. *agr*-deficient *S. aureus* strains cause less severe infections than their wild-type counterparts, highlighting the importance of *agr*-mediated virulence factors in infection [265,266,267]. Several small-molecule ligands that interfere with *agr*-mediated virulence factor production have been reported (Figure 7). *agr* inhibitors target signaling downstream of AgrA and AgrC. For instance, solonamide B, a cyclodepsipeptide produced by *Photobacterium halotolerans* [268], inhibits the binding of AIP to AgrC, leading to the attenuation of δ-toxin production and reduced skin inflammation in a mouse model of inflammatory skin disease [269]. Ambuic acid, a secondary fungal metabolite, inhibits AIP signal biosynthesis in *S. aureus* and MRSA in a dose-dependent manner [270,271]. Savirin, a lipophilic drug targets *S. aureus* AgrA by interfering with its transcriptional regulation and exhibits reduced dermatonecrosis in a mouse infection model [272]. However, inhibition of *agr* quorum sensing promotes biofilm formation *in vitro* and reduces biofilm dispersal during osteomyelitis [273,274]. It is, therefore, essential to test antivirulence agents targeting *agr* in the context of non-biofilm and biofilm infection types since inhibiting the *agr* quorum sensing system may diminish toxin production at the risk of supporting infection persistence [275].

## 6. Conclusions

To develop new therapies, it is crucial to know the mechanisms of antibiotic action and the bacterial elements that are potential targets for antibiotics. Promising targets of antibiotics are gene products that are involved in essential bacterial biochemical processes. *S. aureus* cell death induced by antibiotic treatment results from the alteration in one or more essential bacterial cellular components, such as cell wall, cell membrane, ribosome, DNA gyrase, RNA polymerase, and metabolic process folic acid biosynthesis. Furthermore, teichoic acid, aminoacyl-tRNA synthetases, lipid II cycle, auxiliary factors of β-lactam resistance, two-component systems, and *agr* quorum sensing systems are key components of cellular processes and have emerged as promising drug targets for the treatment of *S. aureus* infections. A greater understanding of the core bacterial processes that are targeted by antibiotics, the associated cellular responses, and the antibiotic mechanisms of action would aid in the design of new therapeutics against antibiotic-resistant pathogens.

## Figures and Tables

**Table 1:** Antibiotics currently in global clinical development with expected activity against *Enterococcus faecium*, *Staphylococcus aureus*, *Klebsiella pneumoniae*, *Acinetobacter baumannii*, *Pseudomonas aeruginosa* and *Enterobacter species* (ESKAPE) pathogens. Antibiotics with potential use to treat *S. aureus* infections are shown here.

Drug Class and Agent	Primary Target	Potential Use
Glycopeptide-β-lactam hybrid		
Cefilavancin	Peptidoglycan chain elongation + PBP	ABSSSI
Triazaacenaphthylene		
Gepotidacin	Type IIA topoisomerase	UTI
Benzofuran naphthyridine		
Afabicin	FabI	SSSIs, Bone and joint infections
Defensin mimetic		
Brilacidin	Cell membrane	ABSSSI
Fluoroquinolone		
Finafloxacin EMROK	Type II topoisomerase Type II topoisomerase	ABSSSI, UTI ABSSSI, HA pneumonia
Macrolide		
Nafithromycin	50S ribosome subunit	CA pneumonia
Quinolone		
Taigexyn	Type II topoisomerase	ABSSSI, CA pneumonia
Rifamycin-quinolone hybrid		
TNP-2092	RNA polymerase, DNA gyrase, DNA topoisomerase IV	ABSSSI, BSI
Aminoglycoside		
Apramycin	30S ribosome subunit	BSI, Complicated UTI, HA pneumonia
Benzyl pyridinone		
CG-549	FabI	ABSSSI
Oxazolidinone		
Delpazolid Contezolid/contezolid acefosamil	50S ribosome subunit 50S ribosome subunit	Gram-positive infections (Specific use unclear) ABSSSI
Tetracycline		
KBP-7072 TP-271	30S ribosome subunit 30S ribosome subunit	CA and HA pneumonia CA pneumonia
Benzamide		
TXA709/ TXA707	FtsZ (Cell wall division)	ABSSSI
Cephalosporin + Diazabicyclooctane		
WCK 5222 (Cefepime + Zidebactam)	PBP + β-lactamase	Complicated UTI, HA pneumonia
Cephalosporin + Cyclic boronate		
Cefepime + Taniborbactam	PBP + β-lactamase	Complicated UTI

Source: The Pew Charitable Trusts, Antibiotics currently in global clinical development, March 2021. Retrieved 12 March 2021. SSSIs: skin and skin structure infections, CA: community-acquired, HA: hospital-acquired, UTI: urinary tract infection, ABSSSI: acute bacterial skin and skin structure infections, BSI: bloodstream infections.

**Table 2:** Targets of antibiotics in *S. aureus* and their mechanisms of action.

Antibiotic Class and Agent	Primary Target (Specific Target)	Net Effect	Mechanism of Action
β-lactams Oxacillin	Cell wall synthesis (PBPs)	Peptidoglycan damage Destruction of cell membranes	Oxacillin covalently binds to PBPs, thereby inhibiting the transpeptidase activity of PBP required for bacterial cell wall synthesis [2,86]. This decreases the integrity of the bacterial cell wall and, ultimately, cell death through autolysis.
Cephalosporins Ceftaroline	Cell wall synthesis (PBP2a transpeptidase)	Conformational changes in PBPs	Ceftaroline is a novel β-lactam broad-spectrum cephalosporin that binds to PBPs, including PBP2a, in MRSA with high affinity, thereby inhibiting cell wall synthesis [87,88]. The 1,3-thiazole ring attaches to the 3-position of the cephalosporin nucleus, while the oxime group in the C7 acyl moiety confers enhanced lethality against MRSA [89,90].
Glycopeptides Vancomycin Teicoplanin	Cell wall synthesis (MurNac-pentapeptide, Transglycosylase)	Peptidoglycan damage Destruction of cell membranes	Vancomycin forms hydrogen bonds with the D-Ala-D-Ala termini moieties of the peptidoglycan precursor lipid II, leading to a conformational alteration that prevents incorporation of NAM/NAG peptide subunits into the growing peptidoglycan chain and consequent transpeptidation [91,92]. This alters bacterial cell membrane integrity and increases permeability, leading to bacterial death [3].
Lipopeptides Daptomycin	Cell wall synthesis Cell membrane (Note: the precise mechanism of action has not been established, and a specific molecular target has not been identified)	Destruction of cell membranes	Daptomycin-Ca^2+^ complex targets cell wall biosynthesis in *S. aureus* by forming a tripartite complex with undecaprenyl-coupled intermediates and membrane lipids [93]. Daptomycin-Ca^2+^ complex oligomerizes to form micelles [94], which penetrate the cell wall and insert into the cytoplasmic membrane by binding to phosphatidylglycerol head groups [95,96]. This causes membrane depolarization, permeabilization, K^+^ ions leakage, and rapid cell death [97,98].
Streptogramins quinupristin/dalfopristin	Protein synthesis (50S ribosome subunit)	Inhibition of protein synthesis	Dalfopristin binds to 23S ribosomal RNA (rRNA) in the 50S ribosome subunit, causing confirmational change, which increases the binding of quinupristin and results in inhibition of peptidyl transfer [99,100]. Quinupristin binds to a nearby site on the 50S ribosome, preventing elongation of polypeptide and causes incomplete chain release. (Note: Each antibiotic alone is bacteriostatic, while their combination shows bactericidal activity)
Aminoglycosides Arbekacin	Protein synthesis (30S ribosome subunit)	Inhibition of protein synthesis	Arbekacin binds to four nucleotides of 16S rRNA and single amino acid of protein S12, thereby interfering with the decoding center of the bacterial 30S ribosome subunit [101]. This leads to inaccurate induction and inhibition of translation, preventing protein synthesis [102,103,104].
Oxazolidinones Linezolid Tedizolid	Protein synthesis (70S ribosome by linezolid 50S ribosome by tedizolid)	Inhibition of protein synthesis	Linezolid inhibits the initiation of ternary complex formation between *N*-formylmethionyl-tRNA (tRNA^fMet^), mRNA, and the 70S ribosome, resulting in the inhibition of bacterial protein synthesis [4]. Tedizolid binds to 23S rRNA of the 50S ribosome subunit, thereby preventing the formation of the 70S ribosomal initial complex, resulting in the inhibition of bacterial protein synthesis [5,105].
Macrolides Azithromycin	Protein synthesis (50S ribosome subunit)	Inhibition of protein synthesis	Azithromycin interacts with bacterial 23S rRNA on the 50S ribosome subunit and inhibits translation by targeting aminoacyl-tRNA, peptidyl-tRNA, or the peptide exit tunnel [6].
Tetracyclines Tetracycline	Protein synthesis (30S ribosome subunit)	Inhibition of protein synthesis	Tetracycline interacts with the bacterial 30S ribosome subunit, preventing the binding of aminoacyl-tRNA (aa-tRNA) to the A site, resulting in inhibition of bacterial protein synthesis [106,107].
Rifamycins Rifampicin (rifampin)	Nucleic acid (RNA) synthesis (RNA polymerase)	Inhibition of protein synthesis Destruction of cell membranes	Rifampicin inhibits bacterial DNA-dependent RNA polymerase, resulting in the suppression of RNA synthesis and bacterial cell death [9,10]. Rifampicin binds in a pocket of the RNA polymerase β subunit within the DNA/RNA channel, preventing transcription by blocking elongation of the 5′ end of the RNA transcript, thus inhibiting protein synthesis [10]. (Note: Rifampicin retains bactericidal activity against non-growing bacterial cultures)
Fluoroquinolones Delafloxacin	Nucleic acid (DNA) synthesis (DNA gyrase and DNA topoisomerase IV)	Damage to DNA replication Destruction of chromosome	Delafloxacin targets two bacterial enzymes: DNA topoisomerase II (DNA gyrase) and DNA topoisomerase IV. Control of supercoiling within treated cells is lost, resulting in impaired DNA replication [7]. Generally, topoisomerase IV is the preferred target in Gram-positive bacteria, whereas DNA gyrase is the preferred target in Gram-negative bacteria [8].
Sulfonamides Sulfadiazine	Folic acid biosynthesis (Dihydropteroate synthase)	Inhibition of nucleic acid (DNA) synthesis Inhibition of cell division	Sulfadiazine acts as a competitive inhibitor of dihydropteroate synthase [108], an enzyme that reduces p-aminobenzoic acid (PABA) to form dihydropteroate in the folic acid biosynthesis pathway [109,110], resulting in a slow-acting bacteriostatic effect [111].
Sulfamethoxazole-Trimethoprim Co-trimoxazole	Folic acid biosynthesis (Dihydropteroate synthase by Sulfamethoxazole (SMX) and dihydrofolate reductase by trimethoprim (TMP))	Inhibition of nucleic acid (DNA) synthesis Inhibition of protein synthesis	SMX inhibits dihydropteroate synthase, leading to the inhibition of folic acid biosynthesis, while TMP binds and inhibits dihydrofolate reductase, preventing the conversion of dihydrofolic acid to tetrahydrofolate [11,112]. The SMX–TMP combination acts synergistically to block two consecutive steps in nucleic acid and protein biosynthesis [113].

## Data Availability

Not applicable.
